# The endotracheal tube microbiome associated with *Pseudomonas aeruginosa* or *Staphylococcus epidermidis*

**DOI:** 10.1038/srep36507

**Published:** 2016-11-04

**Authors:** An Hotterbeekx, Basil B. Xavier, Kenny Bielen, Christine Lammens, Pieter Moons, Tom Schepens, Margareta Ieven, Philippe G Jorens, Herman Goossens, Samir Kumar-Singh, Surbhi Malhotra-Kumar

**Affiliations:** 1 Laboratory of Medical Microbiology, University of Antwerp, Wilrijk, Belgium; 2 Vaccine & Infectious Disease Institute, University of Antwerp, Wilrijk, Belgium; 3 University of Antwerp, Wilrijk, Belgium; 4 Molecular Pathology group, Cell Biology and Histology, University of Antwerp, Wilrijk, Belgium; 5 Critical Care Unit, Antwerp University Hospital, Edegem, Belgium; 6 Antwerp University Hospital, Edegem, Belgium

## Abstract

Ventilator-associated pneumonia (VAP) is one of the commonest hospital-acquired infections associated with high mortality. VAP pathogenesis is closely linked to organisms colonizing the endotracheal tube (ETT) such as *Staphylococcus epidermidis* and *Pseudomonas aeruginosa*, the former a common commensal with pathogenic potential and the latter a known VAP pathogen. However, recent gut microbiome studies show that pathogens rarely function alone. Hence, we determined the ETT microbial consortium co-colonizing with *S. epidermidis* or *P. aeruginosa* to understand its importance in the development of VAP and for patient prognosis. Using bacterial 16S rRNA and fungal ITS-II sequencing on ETT biomass showing presence of *P. aeruginosa* and/or *S. epidermidis* on culture, we found that presence of *P. aeruginosa* correlated inversely with patient survival and with bacterial species diversity. A decision tree, using 16S rRNA and patient parameters, to predict patient survival was generated. Patients with a relative abundance of *Pseudomonadaceae* <4.6% and of *Staphylococcaceae* <70.8% had the highest chance of survival. When *Pseudomonadaceae* were >4.6%, age of patient <66.5 years was the most important predictor of patient survival. These data indicate that the composition of the ETT microbiome correlates with patient prognosis, and presence of *P. aeruginosa* is an important predictor of patient outcome.

Assisted ventilation is performed through an endotracheal tube (ETT), that is readily colonized by bacteria within 24 hours after intubation^1^,^2^. These microorganisms, migrating along the ETT cuff and inside the lumen of the ETT, form a so called biofilm, together with a network of secretions. Biofilm formation is indeed facilitated by the presence of nutrient-rich patient material which accumulates in the lower part of the ETT^3^.

Ventilator associated pneumonia (VAP) occurs in approximately 9–27% of all mechanical ventilated patients after at least 48 h of ventilation and is associated with high mortality and morbidity^4^,^5^. The exact mechanisms leading to the development of VAP are not understood, although a strong link exists with the microbial consortium on the ETT^1^,^6^,^7^. Endotracheal secretions often mirror the microbial consortium present in the ETT, at least at the resolution achieved by culture methods^8^,^9^. For this reason it is believed that micro-aspiration of detached pieces of biofilm containing harmful organisms might cause infection in the lower respiratory tract, and are believed to significantly contribute to the occurrence of (VAP)^1^. Regular screening of endotracheal secretions facilitates early diagnosis of the microorganisms linked to VAP and has been shown to impact on patient treatment and survival^10^.

*Pseudomonas aeruginosa* is one of the most common causes of VAP followed by Gram positive organisms like *Staphylococcus aureus*^5^. In some cases, however, no clear pathogen can be detected by conventional culture methods^11^, while in other cases a significant growth of oro-pharyngeal or cutaneous commensals including *Staphylococcus epidermidis* can be observed, suggesting that these organisms are not as harmless as frequently believed^12^. Recently, the presence of oro-pharyngeal microorganisms was demonstrated on the ETTs of mechanically ventilated patients^6^ and in respiratory samples^11^, however, the presence of any of these organisms on the ETT did not always lead to VAP with these bacteria^7^,^12^, indicating a contribution from host factors and the general health status of the patient. In fact, an important role for the entire bacterial consortium on the ETT in the development of VAP has been suggested^6^ and involves interspecies interactions that provide nutrient sources or produce growth inhibiting molecules^13^,^14^. While the presence of specific bacteria leading to dysbiosis has been shown for diseases like periodontitis and cystic fibrosis^15^,^16^, there is a lack of knowledge of such relationships within ETT biofilms and their potential role in the development of VAP.

Next generation sequencing technology is a powerful molecular tool to detect a wide variety of species, including the unculturable fraction.

By screening a large collection of ETTs obtained from mechanically ventilated patients at the Antwerp University Hospital over a three-year period, we selected 39 ETTs that showed positive culture results for *P.* aeruginosa, a major VAP pathogen, and *S. epidermidis*, the most frequently cultured bacterium in this study. We aimed (i) to study organism and species diversity in ETT biofilms using a culture independent metagenomic approach by bacterial 16S rRNA and fungal internal transcribed spacer (ITS) amplicon sequencing, (ii) to determine the core microbiomes on the ETTs which were culture positive for the key organisms *P. aeruginosa* and *S. epidermidis* and (iii) to link these findings with patient parameters in an attempt to identify markers of disease or of patient outcome.

## Results

### Abundance of ETT biomass does not correlate with VAP aetiology or patient prognosis

**Figure 1:**
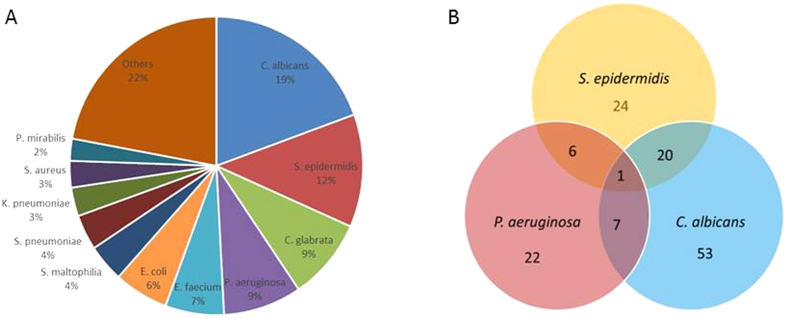
Culture results of 203 ETTs collected during the study (**A**) and of ETTS showing presence of *P. aeruginosa, S. epidermidis* and *C. albicans* (**B**).

To study if the ETT biomass quantity or composition correlate to the development of VAP or to patient prognosis, we prospectively collected 203 ETTs from mechanically ventilated patients admitted to the intensive care unit (ICU) during a 3-year period, from April 2011 until December 2013, at the University hospital Antwerp, Belgium. Of these 203 patients, 44 progressed to pneumonia (VAP). Previous studies have shown that *P. aeruginosa* is a common VAP pathogen associated with high mortality and morbidity^5^ whereas *S. epidermidis* is the most frequently isolated bacterium in this study (51/203 ETT) that under specific circumstances can become a nosocomial pathogen^12^. *P. aeruginosa* was cultured from 36/203 ETT and was the second most common bacterium (Fig. 1A). To further study co-carriage with *P. aeruginosa or S. epidermidis*, ETTs from 39 patients showing presence of *P. aeruginosa* (n = 13), *S. epidermidis* (n = 21), or both (n = 5) on culture were selected for further analysis. Fifteen of these 39 patients progressed to pneumonia (VAP). In the ETTs showing presence of *P. aeruginosa*, this organism was also identified as the aetiology of the 4 VAP cases in broncho-alveolar lavage (BAL) cultures of these patients. However, in ETTs showing presence of *S. epidermidis*, other organism(s) were identified as the aetiological agent of VAP (9/11) or as a non-*aureus Staphylococcus* (1/11) or remained unidentified (1/11). Summary of the data from all 39 patients is shown in Table 1 and details on patients progressing to VAP in Supplementary Table S1.

**Figure 2:**
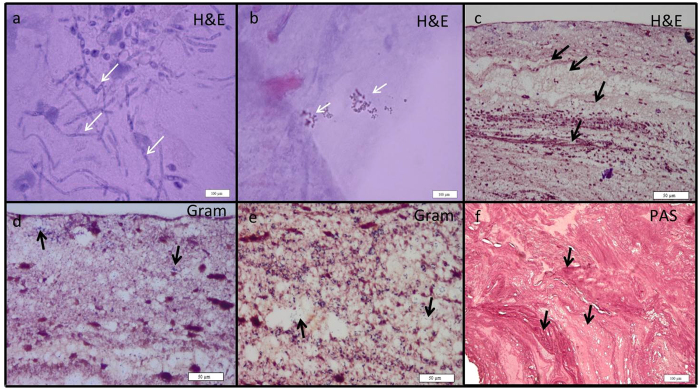
Histology of typical ETT biofilms. H&E staining revealed the presence of candida hyphae, white arrows (**a**); bacterial clusters, white arrows (**b**); and a clearly layered structure, black arrows (**c**); lacunae that harbored human cells are shown in the Gram-stained slices (**d** and **e**, black arrows); PAS stain also showed the layered structure (black arrows) and many cavities (**f**).

Biomass was visually scored under a 5x microscope as present or absent and validated by histology (Fig. 2). H&E staining revealed a typical laminar patterning in the ETT biomass associated with small clusters of bacteria or yeast hyphae. We also found many lacunae in the structures that harboured human cells. Similar structures have been previously observed by Inglis *et al*.^17^ who performed light and confocal scanning laser microscopy on ETT biofilms. Light and fluorescence microscopy allow the preservation of the ETT biofilm structures, in contrast to scanning electron microscopy used in most studies^2^,^9^,^18^ were the original structure is lost.

In our study, the presence of visible biomass in the ETT lumen did not correlate with development of VAP (P = 0.104) or patient survival (P = 0.100), indicating that the amount of ETT biofilm is not a major effector of VAP or patient prognosis. During mechanical ventilation, endotracheal secretions are removed on a regular basis with a suction catheter to prevent biofilm formation and VAP^19^. Furthermore, the ETT biofilm mainly consists of patient material in addition to bacteria and the exact amount of secretions produced is patient dependent^17^. These findings suggest that the presence of biomass as such is not necessarily bad for the patient prognosis and composition might play a role as well.

In addition, the APACHE II scores also did not significantly differ among the patients that developed VAP or not (P = 0.943) or survived or not (P = 0.196), indicating that the physiological condition was the same at the time of intubation. This result is confirmed by k-means clustering and logistic regression analysis which showed no correlation between patient survival and the reason of admission to the ICU. These data raised the question what other effectors, like the microbial consortium on the ETT or other patient related parameters are likely to play a role in VAP aetiology and patient prognosis.

### A two-step cluster analysis reveals three natural clusters with patient survival and development of VAP as the two main cluster predictors

To investigate patient-related parameters, the 39 patients were subdivided by a two-step cluster analysis, which facilitates the discovery of natural clusters of individuals with similar characteristics within a larger population^20^. The 39 patients were divided into three clusters with patient survival upon extubation (predictor importance 1) and development of VAP (predictor importance 0.55) being the most important cluster predictors (Table 2). All patients in clusters 2 and 3 (n = 16 and 12, respectively) survived whereas all in cluster 1 (n = 11) died. Details of the patients are shown in Supplementary Table S1. Clusters 2 and 3 are differentiated by the development of VAP; 13 of the 16 patients (81.2%) in cluster 2 and none of the 12 in cluster 3 progressed to VAP. On the other hand, only 2 patients from cluster 1 (18.2%), wherein 100% mortality was observed, developed VAP. Remarkably, all patients in cluster 2, who showed the highest incidence of VAP, survived. Because the development of VAP is usually known to be associated with high mortality^5^, a combination of other factors might determine survival in this subset of patients. The third most important predictor of clustering was the presence of a visible biomass on the ETT upon extubation (predictor importance 0.25). All ETTs of cluster 3 and 9/11 (81.8%) ETTs of cluster 1 showed a visible biomass, while in only half of the ETTs of cluster 2 a biomass was visible.

As a fourth predictor, *P. aeruginosa* was more frequently isolated from ETTs in cluster 1 (7/11), while *S. epidermidis* was more frequent in cluster 2 (9/16 alone and 5/16 with *P. aeruginosa*) and cluster 3 (8/12) (predictor importance 0.175). Average APACHE II scores (predictor importance 0.170) were similar for cluster 1 (26.55) and cluster 2 (25.5) and lower for cluster 3 (18.50), where all patients survived and none developed VAP. A least significant difference (LSD) post-hoc analysis showed that survivors in cluster 2 were on average 10 years younger than the patients who died in cluster 1 (P = 0.043), while there was no significant difference in age compared to cluster 3.

### The microbiome present on the ETTs correlates with patient prognosis

Next, we questioned whether the microbial composition of the ETT biomass might correlate to patient prognosis. Therefore, 16S rRNA analysis was performed to identify the ETT microbial consortium co-existing with *P. aeruginosa* or/and *S. epidermidis* and subjected these data together with the patient parameters to a multivariate analysis.

**Figure 3:**
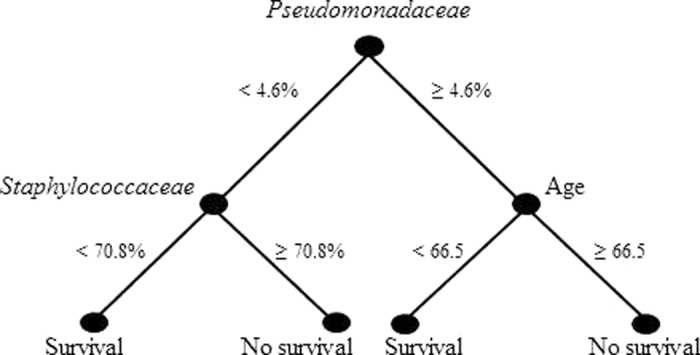
Decision tree predicting patient survival.

First, we generated a decision tree that identified a low relative abundance of family *Pseudomonadaceae* as the key predictor of patient survival (Fig. 3). A relative abundance of *Pseudomonadaceae* below 4.6% and of *Staphylococcaceae* below 70.8% correlated with a high chance of patient survival. When the relative abundance of *Pseudomonadaceae* was above 4.6%, patient age below 66.5 years also correlated with a higher chance of survival, confirming the importance of the organisms present on the ETTs and age of the patient (Fig. 3).

**Figure 4:**
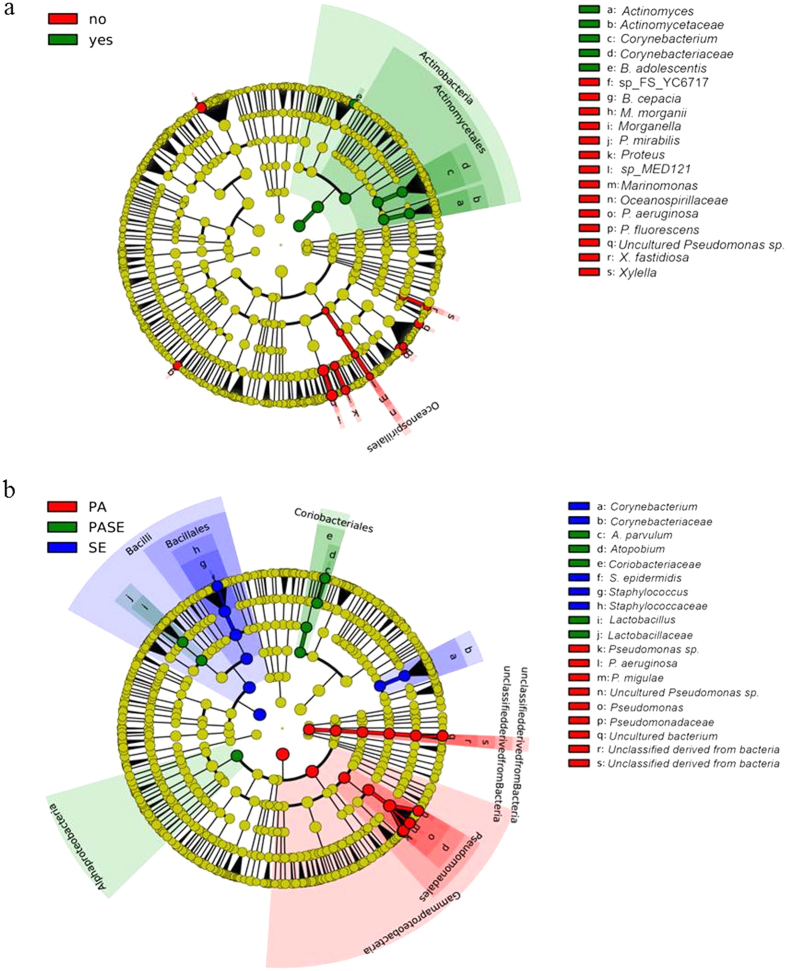
Cladogram showing the association of distinct ETT microbial components (OTUs) with patient survival (**a**) or with the 3 groups of ETTs defined by culture (**b**). The inner circles represent the highest taxonomic level and the coloured bands contain the taxonomic levels which are significantly more associated with one group of patients (LDA score > 2). The cladogram was generated using the LefSe galaxy tool. A. Red: patients that did not survive upon extubation; Green: patients that survived upon extubation. B. Red: ETT with positive culture for *P. aeruginosa*; blue: ETT with positive culture for *S. epidermidis*; green: ETT with positive culture for both *P. aeruginosa* and *S. epidermidis.*

Second, we investigated whether other members of the ETT microbiome could be associated with patient survival using the online binomial tool LefSe dedicated for biomarker discovery^21^. This tool considers frequency as well as relative microbial abundance and performs a linear discriminant analysis (LDA) to calculate the effect size of each parameter^21^. An LDA score above 2 was considered significant. LefSe analysis showed that ETTs of patients who survived (n = 28) were more likely to harbour the phylum *Actinobacteria* (Fig. 4a, n = 11, LDA score of 4.8). Within this phylum, *Actinomyces* and *Corynebacterium* were more frequently identified in the ETTs of the surviving patients (18/28 and 15/28, respectively) compared to the patients that did not survive (1/11 and 4/11, respectively; LDA score >3.6). In addition, *Bifidobacterium adolescentis* was identified in 9/28 ETTs of the survivors while it was completely absent in the ETTs of the patients that died (LDA score >2.4). Furthermore, the ETTs of patients that died showed a close association with the genus *Pseudomonas*, including *P. aeruginosa* (8/11)*, P. fluorescens* (3/11) and uncultured *Pseudomonas spp*. (6/11). Also noteworthy was the presence of *Morganella morganii* (4/11), *Burkholderia cepacia* (2/11), *Proteus mirabilis* (2/11), *Marinomonas* sp. MED121 (2/11), and *Xylella fastidosa* (2/11) on the ETT of the patients that died. While these were found in only a fraction of the ETTs from the non-surviving patients, their frequency and abundance was significant in this group compared to the ETTs of the survivors (*Morganella morganii* 2/28, others absent, LDA score >2).

### Distinct predominant microbiomes are associated with the presence of *P. aeruginosa* and *S. epidermidis*

After we identified significant associations between microbial composition and patient prognosis, we also questioned whether a microbial fingerprint might be linked to development of VAP. As the aetiology of VAP differed between the three groups of ETTs identified by culture (Supplementary Table S1), we aimed to identify the differential microbiomes of these three groups in correlation to VAP as well as a common ETT core microbiome.

**Figure 5:**
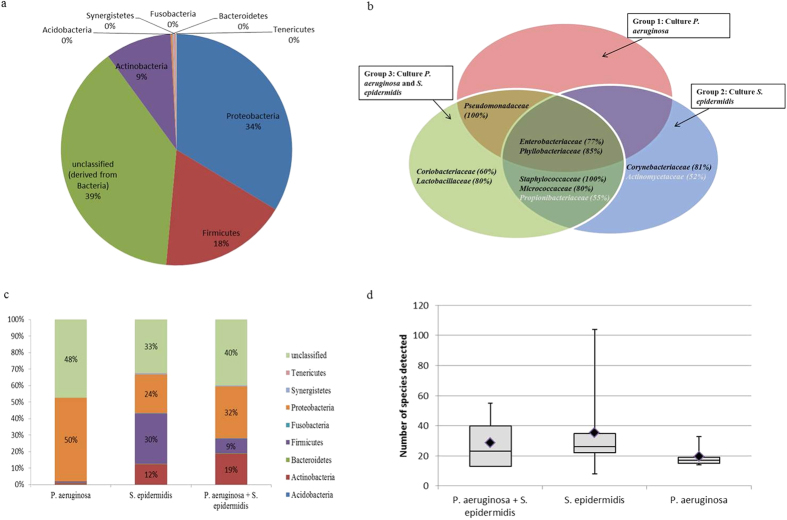
Distribution of the relative abundance of the major phyla. The phyla Proteobacteria and Firmicutes were the most abundant. BLAST analysis revealed that approximately 90% of the unclassified sequences also belonged to Proteobacteria (**a**). OTUs defined on the family level found to be associated with the 3 groups of ETTs. Families present in at least 50% of the ETTs in a certain group were assigned to the microbiome of that group. (**b**). Distribution of OTUs, defined at the phylum level, within the 3 groups of ETTs (**c**). Species diversity in the three groups of ETTs. Black squares: average number of species (**d**).

Firstly, classification of all 16S sequences by alignment to the SILVA reference database showed a total of 8 different phyla that were associated with the ETTs analysed in this study. Within these phyla, 69 different families harbouring 354 different species were identified. The majority (61%) of the sequences belonged to three phyla: *Proteobacteria* (34%), *Firmicutes* (18%) and *Actinobacteria* (9%) as shown in Fig. 5a. Of note, 39% of the sequences remained unclassified when compared to existing reference sequences in the Greengenes, SILVA and RDP databases. An additional BLAST against the NCBI nr database revealed that the majority (89%) of these were of the *Proteobacteria* family.

Secondly, we aimed to identify which families were present on the majority of ETTs across the three groups regardless of their relative abundance. *Enterobacteriaceae* and *Phyllobacteriaceae* could be identified on the majority of the ETTs (77% and 86%, respectively) (Fig. 5c). However, at the level of the genus and species, the *Enterobacteriaceae* family showed a large diversity. The genera *Escherichia* and *Klebsiella* were the most and second-most commonly identified with *E. coli*, *E. albertii* and *K. pneumoniae* as the main representatives. However, none of these species were present in more than 50% of all ETTs. In contrast to the high variability of the *Enterobacteriaceae*, the *Phyllobacteriaceae* had only one representative, *Phyllobacterium myrsinacearum*, which was present at low abundance in 86% of all ETTs. More details on the species distribution are given in Supplementary Table S2.

Thirdly, we identified the phylum distribution and species diversity in the three groups of ETTs (Fig. 5b). In the ETTs of the *P. aeruginosa* group, the phylum *Proteobacteria* was most abundant (98%), while *Firmicutes* and *Actinobacteria* (1% each) were present in a minority. In contrast, ETTs belonging to the *S. epidermidis* group showed a more even distribution over the three phyla: *Proteobacteria* (56%), *Firmicutes* (30%) and *Actinobacteria* (12%). In the third group, the majority of sequences were also assigned to the *Proteobacteria* (72%), followed by *Actinobacteria* (19%) and *Firmicutes* (9%). At lower taxonomic levels, ETTs belonging to the *S. epidermidis* group showed the maximum species diversity (290 species in total) and the highest average number of species per tube (35 species, 3357 reads). This was in contrast to the ETTs of the *P. aeruginosa* group that harboured on average 20 different species (3791 reads) per tube and in total 116 different species (P = 0.029) (Supplementary Table S3).

Lastly, we used the LefSe tool to discover distinct microbial signatures associated with each of the three ETT groups (Fig. 4b). In the *P. aeruginosa* group, only bacteria belonging to the family *Pseudomonadaceae* were identified and these included various *Pseudomonas spp.* These data provide *in vivo* evidence for previous laboratory based studies describing the production of antibacterial factors and inhibition of competitive colonizers (non-pseudomonads) both on co-cultures and in a biofilm phenotype by *P. aeruginosa*^22^,^23^,^24^,^25^,^26^,^27^. In the *S. epidermidis* group, *Corynebacteriaceae* were identified in 17/21 (80%) of the ETTs (Fig. 5c). Since the VAP aetiology was variable in this group, differences in the ETT microbial signatures of patients developing VAP or not were sought. Interestingly, presence of *Klebsiella pneumoniae* (7/9 VAP, 2/12 non-VAP) and *Serratia marcescens (*8/9 VAP, absent in non-VAP) among the *S. epidermidis* group that developed VAP was highly significant (LDA > 4). Finally, in the group of ETTs containing both *P. aeruginosa* and *S. epidermidis* (n = 5), *Lactobacillaceae* (4 ETTs) and *Coriobacteriaceae* (3 ETTs) were more prevalent (Figs 4b and 5c). The family of the *Lactobacillaceae* showed mainly *Lactobacillus* but sequences could not be identified to the species level. *Atopobium parvulum* was the main representative of the *Coriobacteriaceae.* We can conclude that the microbial composition of the ETTs consists of two parts: a common (up to family level) microbiome shared by all ETTs and a clearly distinct microbiome at least for the ETTs harbouring *P. aeruginosa* or *S. epidermidis*. Importantly, our finding that presence of *P. aeruginosa* (and lower species diversity) correlates strongly with worse patient outcome underlines the importance of detecting its presence as a marker of patient prognosis.

### *Candida spp.* are the most common fungi in the ETT microbiome

During culture of the ETTs, *Candida albicans* and *Candida glabrata* were the most and third most common organisms isolated, with an incidence of 81/203 and 37/203 ETTs, respectively (Fig. 1A,B). In the selection of ETTs used for this study, *Candida* spp. were present in 17/39 tubes, of which 11 also showed the presence of *S. epidermidis,* 4 of *P. aeruginosa* and 2 of both. ITS sequencing identified 55 different fungal species, of which the majority (70%) belonged to the phylum *Ascomycota* and a smaller proportion (27%) to *Basidiomycota*. All 17 samples harboured at least one and in most cases two of the following *Candida* species: *C. albicans* (11 ETTs)*, C. glabrata* (13 ETTs) or *C. tropicalis* (14 ETTs). Additional *Candida* species identified include *C. catenulata* (8 ETTs)*, C. dubliniensis* (2 ETTs) and *C. parapsilosis* (1 ETT) (Supplementary Table S3).

Furthermore, LefSe analysis assigned the family *Onygenaceae* to the group of ETTs from VAP patients with an LDA score of 2.5, however, this association was only present at order and family level, probably due to the low number of VAP patients in this subgroup (4 ETTs). Of note, relative abundance of strict and facultative anaerobes was significantly higher in the ETTs harbouring *Candida spp.* compared to the ETTs without *Candida* (54% versus 43% relative abundance, respectively; strict anaerobes 13% versus 5%; facultative anaerobes 41% versus 38%, respectively) (Z-test, P < 0.001). These differences between the two groups were primarily due to relative proportions rather than species composition except for the genus *Prevotella*, which was found to be significantly associated with presence of *Candida* by LefSe analysis (LDA < 2). Prior studies have shown that fungi are important for maintaining oral microbiome homeostasis but their role in the ETT biofilm remains unclear^28^,^29^. Remarkably, in our samples, *Candida* spp. were more frequent in the ETTs harbouring *S. epidermidis* and were only rarely co-isolated with *P. aeruginosa* (Fig. 1B). These results support earlier findings that *P. aeruginosa* and *C. albicans* share an antagonistic relationship^30^ whereas *Staphylococci* and *C. albicans* are thriving well together^31^.

## Discussion

This study, to our knowledge, is the largest to date analysing the ETT biofilm in ventilated patients. Instead of a random molecular profiling of the bacterial community in the ETT, we opted to study the accessory microbiome co-colonizing with *P. aeruginosa* or *S. epidermidis*, the former is one of the most common causes of VAP and the latter one of the most common commensals with a known pathogenic potential. Both are the most common bacteria cultured from the ETTs in this study. Our aim was to decipher consistent associations between presence of *P. aeruginosa* or *S. epidermidis* and other organisms in the ETT biofilm in correlation with multiple patient parameters. Finally, this study provides a proof-of-concept of utilization of 16S microbiome data and patient parameters to generate a prognostic algorithm to predict patient survival.

We found that *P. aeruginosa* was consistently present in patients that did not survive upon extubation, suggesting that it might be an indicator of a worse patient outcome. Multivariate analysis of 16S data and patient parameters showed that relative abundance of *Pseudomonadaceae* was the most important predictor of patient survival followed by age and relative abundance of *Staphylococcaceae*. Our identified best-case scenario for patient survival was an abundance of *Pseudomonadaceae* below 4.6% and of *Staphylococcaceae* below 70.8%. When the relative abundance of the *Pseudomonadaceae* was above 4.6%, a patient age <66.5 years became the most important predictor of patient survival.

Although this algorithm is based on ETT microbiome data and represents a later stage of microbial colonization, previous research has shown consistent correlations between organisms isolated from ETTs and earlier stage respiratory samples like endotracheal aspirates and BALs^8^,^18^. In the present study, for patients who developed VAP due to *P. aeruginosa* (BAL cultures and clinical diagnosis), we were also able to identify the pathogen in the ETT of all patients using cultures and 16S analysis. Therefore this algorithm would be a useful addition to current ICU protocols in hospitals performing routine endotracheal aspirate sentinel cultures as well as for clinical trials pre-enriching for patients colonized with *Pseudomonas aeruginosa* and likely to develop VAP.

In addition to *P. aeruginosa*, the microbiome of patients who did not survive harboured other potentially pathogenic bacterial genera such as *Morganella*, *Proteus*, *Burkholderia* etc (Fig. 4a). LefSe analysis showed that not all genera were consistently present in all patients who died, however, there was a clear difference with the microbiome of surviving patients. The ETT microbiome of the latter group of patients tended to harbour the phylum *Actinobacteria,* which include *Bifidobacteria*, commonly used as probiotics, and non-diptheriae *Corynebacteriaceae*. Interestingly, a recent study analysing the benefits of probiotic *Bifidobacterium longum* administration in patients undergoing surgery, showed that an increase in relative abundance of *Actinobacteria* in the gut correlated inversely with blood inflammatory parameters^32^. These data support our findings that surviving patients tend to harbour a ‘healthy’ microbiome. Whether microbial shifts or dysbiosis in the endotracheal microbiome are the cause or the consequence of a worse patient outcome remains to be investigated and would require sequential sampling of the endotracheal microbiota in order to obtain better insight on the community dynamics.

Loss of microbial diversity has been previously identified as a consistent marker of a diseased microbiome^33^,^34^. In our study, while the incidence of VAP was indeed lower in the *S. epidermidis* colonized group, the patients who did develop VAP in this group did not show any loss of microbial diversity compared to others in this group (p = 0.464). In fact, we found a significant loss of microbial diversity to be associated with *P. aeruginosa* colonization. This competitive exclusion is a hallmark of *P. aeruginosa* colonization and has also been previously observed in cystic fibrosis^26^. These data are also supported by *in vitro* studies demonstrating the predominant nature of *P. aeruginosa* and its inhibition of other potential pathogens or colonizers^13^,^22^,^25^,^26^. Of note, unlike the *P. aeruginosa* colonized group, the high microbial diversity in the *S. epidermidis* colonized group was also reflected in the concurrent difficulty in identifying a clear VAP pathogen in BAL cultures. While the possibility exists that the causative pathogen is a nonculturable (in the culture conditions utilized) organism, VAP might also be caused by a microbial consortium without a predominant pathogen^14^. Finally, lack of a clear VAP pathogen in BAL might also be due to the fact that microbiology laboratories conventionally report only the predominant organism (in most cases a *Staphylococcus* in the *S. epidermidis* group), while a low abundance pathogen might be missed on culture. In the *S. epidermidis* group, we found a consistent co-presence of *K. pneumoniae* and *S. marcescens*, present in relatively low numbers (median: 10.5 and 5 reads respectively) in patients who developed VAP whereas both were absent in the patients that did not develop VAP. Of note, ETT cultures did not grow *K. pneumoniae* and *S. marcescens* and their presence was only detected upon 16S sequencing. Since the ETT reflects the end-stage microbiome, the potential role of these correlations remain to be validated *in vitro* and in earlier stage patient samples.

Finally, *Candida spp.*, which are known nosocomial pathogens, were more often isolated from ETTs of the *S. epidermidis* group (13/17). These results corroborate prior studies demonstrating the antifungal activity of *P. aeruginosa*^31^,^35^,^36^, and the frequent co-isolation of *S. epidermidis* and *C. albicans* in oral polymicrobial infections^13^,^31^,^37^. Fungi are also part of the healthy oral microbiota, supporting the growth of oral anaerobes and increasing the resilience of the consortium^28^,^38^. Indeed, we also found a remarkably higher relative abundance, but not differing species, of anaerobes in ETTs harbouring *Candida* versus those that did not.

In conclusion, this study represents one of the first instances/attempts of utilization of the patient microbiome and clinical parameters to develop a prediction model of patient prognosis. The next steps would involve analysis of sequential respiratory samples in a larger cohort of intubated patients to understand the endotracheal biofilm dynamics and to develop models based on presence or relative abundances of early microbial markers that are predictive of patient prognosis and of the risk of developing VAP.

## Materials and Methods

### Study design and sample collection

ETTs from 203 mechanically ventilated patients admitted to the intensive care unit (ICU) were collected prospectively during 2011–2013 at the Antwerp University Hospital in accordance with relevant regulations and guidelines. Inclusion criteria were minimum 48 hours of ventilation, non-pregnant and age above 18 years. This study was approved by the ethical commission of the Antwerp University Hospital (12/12/112) and informed consent to use these data for research purpose was obtained. The lower 15 cm of the collected ETTs was processed immediately. ETTs were divided into 5 parts, A–E (Supplementary Figure S1). Parts A and E were cultured by gently tapping the ETT pieces on Colombia blood agar and Sabouraud’s dextrose agar. Visually distinct colonies were identified using MALDI-TOF (Bruker Daltonics, Bremen, Germany). Thirty-nine ETTs that yielded *P. aeruginosa* (n = 13) or *S. epidermidis* (n = 21) or both (n = 5) were studied further. From each of the 39 ETTs, parts below the cuff (D, E and E1) were utilized for sequencing (D and E) and for microscopy (E1) (Supplementary Figure S1).

### Microscopic analysis

Part E1 of ETT was fixed in 4% paraformaldehyde for 24 h. When a biomass was visible on the internal lumen of the fixed E1 slices, the mass was removed by scalpel and prepared for standard paraffin embedding as done previously^39^. Histology was performed on 5 μm thick sections stained with Gram, haematoxylin and eosin (H&E), and Periodic acid Schiff (PAS) stains. All light microscopy images were captured on a Zeiss lab.A1 (Heidelberg, Germany) microscope equipped with a UC30 colour camera (Olympus, Antwerp, Belgium).

### DNA extraction and multiplexed pyrosequencing

Adherent material on parts D and E of the ETT was removed by sonication and vigorous vortexing (5 min. each). Samples were centrifuged (5 min. at 17949× g, 5430 R centrifuge, Eppendorf, Rotselaar, Belgium) and reduced to a volume of approximately 0.5 ml. DNA was extracted using the Masterpure complete DNA and RNA purification kit (Epicentre, Leusden, The Netherlands) as recommended with the following modification: 1 μl Ready-lyse lysozyme solution (Epicentre, Leusden, The Netherlands) was added to 150 μl sample followed by incubation at 37 °C for 30 minutes prior to the extraction. Primers V345_341F (CCT ACG GGR SGC AGC AG) and V345_909R (TTT CAG YCT TGC GRC CGT AC) were used for 16S amplification of the V3–V5 region and primers (ITS-F3): GCATCGATGAAGAACGCAGC and (ITS-R4): TCCTCCGCTTATTGATATGC for amplifying the fungal ITS-II region using multiplexed Roche 454 pyrosequencing^40^,^41^,^42^. Samples were purified by bead-beating prior to amplification. Purified and quantified PCR products were pooled in equimolar amounts and uni-directional sequencing on ½ run was performed on 454 GS FLX Sequencer (Roche, Basel, Switzerland) using Titanium FLX reagents, resulting in 5000 reads with a 400–600 bp read length on average per sample (Microsynth, Balgach, Switzerland). Sequence analysis was performed using the online MetaGenome Rapid Annotation using Subsystem Technology (MG-RAST)^43^.

### Sequence analysis

Sequences were sorted based on their multiplex identifier tag and the template-specific primers were trimmed. Sequences shorter than 200 bp were eliminated, and others were aligned against the SILVA database (release 115) using the mothur tool^44^,^45^. A second-round quality filtering eliminated non-aligned reads with <95% alignment and chimera sequences were removed using the UCHIME^46^ tool implemented in mothur. The taxonomic classification was performed using mothur based on a Bayesian method^47^ with K-mer size of 8bp and 1000 replicates. An additional parallel analysis was performed using the online analysis server MetaGenome Rapid Annotation using Subsystem Technology (MG-RAST)^43^. After pre-processing of the raw reads with default parameters, the 16S sequences were processed similar as described above. Processed reads were searched against the SILVA database using BLAT^48^ for rRNA identification with 97% identity to cluster the sequences, where after the longest sequence of each cluster was used as a representative. BLAT similarity search with default parameters was performed against the M5rna database with the max e-value cut-off set to 1 × 10^5^, the min% identity cut-off to 97% and a minimum alignment length cut-off of 15.

### Analysis of microbial composition of the ETT biofilms

ETTs were grouped into those showing presence of *P. aeruginosa*, *S. epidermidis* or both organisms by culture and their microbial composition was analysed to the family level. Presence/absence of a family in each of the three groups was determined and assigned to the microbiome of a certain group with at a cut-off value of present in 50% of the ETT from a certain group. Differences in relative abundance on all taxonomic levels between the three ETT groups were identified by the binomial disclaim tool linear discriminant analysis effect size (LefSe)^21^. First, LefSe determines features (organisms, clades, operational taxonomic units, genes, or functions) most likely to explain differences between groups by coupling standard tests for statistical significance with additional tests encoding biological consistency and effect relevance. Second, linear discriminant analysis (LDA) is used to rank the features differing between the groups based on their effect sizes^21^. All taxonomic levels showing an LDA score above 2 were considered significant. Statistical significance was set at 0.05 and the strategy for multi-class analysis was set all against one.

### Definitions

VAP was identified according to the classical definition as a bacterial pneumonia present in patients with mechanical ventilation for at least 48 hours combined with a new infiltrate on the chest X-ray, signs of infection and detection of a bacterial causative agent^4^,^49^. The severity of illness in the ICU was scored according to the validated Apache II score^39^.

### Statistical analyses

Statistical analyses were performed in IBM SPSS statistics v2.0. Normality was tested using the Shapiro-Wilk test for normality. One-way ANOVA was used for normally distributed data and the Mann-Whitney U test for not normally distributed data. Two-step cluster analysis was performed to discover natural clusters in the dataset using the Schwarz’s Bayesian Criterion (BIC) and outlier treatment was applied using default parameters. Statistical differences between each variable in the clusters were determined using one-way ANOVA for continuous data and χ2 for categorical data in a post-hoc least significant difference (LSD) analysis. Significance level was 0.05 in all statistical analyses. Logistic regression and K-means clustering of the 16S and patient data were performed in Matlab R2015a (MathWorks, Eindhoven, The Netherlands) and R platform v0.99.47 (RStudio, Boston, USA), respectively. Results of the two-step cluster analysis were confirmed by the k-means clustering algorithm on the R platform.

## Additional Information

**Accession codes:** All sequencing data generated for this project were deposited to European Nucleotide Archive (ENA) under study accession number PRJEB16308.

**How to cite this article**: Hotterbeekx, A. *et al*. The endotracheal tube microbiome associated with *Pseudomonas aeruginosa* or *Staphylococcus epidermidis*. *Sci. Rep.* **6**, 36507; doi: 10.1038/srep36507 (2016).

**Publisher’s note:** Springer Nature remains neutral with regard to jurisdictional claims in published maps and institutional affiliations.

## Supplementary Material

Supplementary Information

## Figures and Tables

**Table 1:** Overview of patient and ETT culture data.

	Total N° ETT: 39
Male	26
Female	11
Age	33–83 (median 61)
Days of intubation	2–43 (median 11)
VAP cases	15
Culture *P. aeruginosa*	13 (4 VAP)
Culture *S. epidermidis*	21 (9 VAP)
Culture *P. aeruginosa* and *S. epidermidis*	5 (2 VAP)

39 ETTs were collected from same number of patients. 15 patients developed VAP during their intubation period. P*. aeruginosa* and/or *S. epidermidis* identified from ETT cultures and subsequent MALDI-TOF. Patients who developed VAP in each group are indicated.

**Table 2:** Two-step cluster analysis of patient data.

Variables	Cluster 1 (n = 11)	Cluster 2 (n = 16)	Cluster 3 (n = 12)
Patient survival (%)	0%	100%	100%
Patients developing VAP (n,%)	2, 18.2%	13, 81.8%	0, 0%
Biomass visible on ETT (n,%)	9, 81.8%	8, 50%	12, 100%
ETT culture results (n,%)	*P. aeruginosa (*7, 63.6%)	*P. aeruginosa* (2, 12.5%)	*P. aeruginosa* (4, 33.3%)
	*S. epidermidis* (4, 36.4%)	*S. epidermidis* (9, 56.25%)	*S. epidermidis* (8, 66.7%)
		Both (5, 31.25%)	
APACHE II scores (average)	26.55	25.5	18.5
Age (average in years)	67.82	57.69	60.5
Duration of intubation in days (average)	12.45	13.69	11.42

Variables are listed in order of importance.
