# Supplementary material for: The endotracheal tube microbiome associated with Pseudomonas aeruginosa or Staphylococcus epidermidis
Source: Sci Rep. 2016 Nov 4;6:36507. doi: 10.1038/srep36507 (PMC5095667; doi:10.1038/srep36507)
Supplement: Supplementary Information [file srep36507-s1.docx]

**The endotracheal tube microbiome associated with *Pseudomonas aeruginosa* or *Staphylococcus epidermidis***

An Hotterbeekx^1,3^*, Basil B. Xavier^1,3^*, Kenny Bielen^2,3^, Christine Lammens^1,3^, Pieter Moons^1,3^, Tom Schepens^4^, Margareta Ieven^1,3,5^, Philippe G Jorens^4,5^, Herman Goossens^1,3,5^, Samir Kumar-Singh^2,3^, Surbhi Malhotra-Kumar^1,3^#

^1^Department of Medical Microbiology, ^2^Molecular Pathology group, Cell Biology and Histology, ^3^Vaccine & Infectious Disease Institute, University of Antwerp, Wilrijk, Belgium ^4^Critical Care Unit, ^5^Antwerp University Hospital, University of Antwerp, Edegem, Belgium

### Supplementary figures and tables


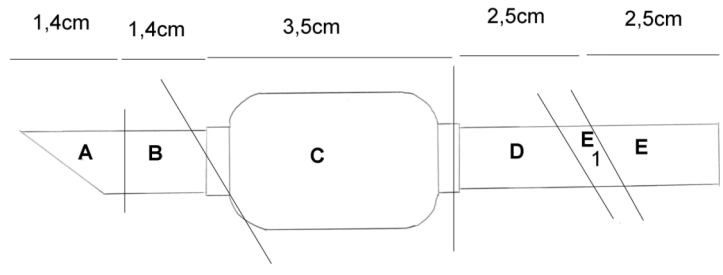


Figure S1: Schematic overview of a typical ETT. Parts A and E were cultured immediately and part D was used for 16S analysis in this study. Part B was stored at -80°C and part E1 was used for microscopy.

Table S1: Clinical data and ETT culture results of all patients. The causative agents were identified in the broncho-alveolar lavage (BAL) samples. If BAL was contra-indicated, an endotracheal aspirate was used.

Table S1: Continued

Table S2: Average relative abundance of the families in the core microbiomes and the percentage of ETTs harbouring them. The ETT are divided in three groups based on culture results.

^a^In the mixed group of *P. aeruginosa* and *S. epidermidis* the family of the *Pseudomonadaceae* could only be detected in 60% of the ETT. The other tubes contained sequences which were assigned to ‘uncultured gammaproteobacterium’ which could not be identified with BLAST.

**Table S3:** Overview of the different *Candida* species identified as a percentage of all ITS reads per ETT.
